# Placenta Accreta Spectrum Disorders: A. Chohan Continuous Squeezing Suture (ACCSS) for Controlling Haemorrhage from the Lower Uterine Segment at Caesarean Section

**DOI:** 10.12669/pjms.39.1.6990

**Published:** 2023

**Authors:** M. Arshad Chohan, Fauzia Butt, Maria Imran, Sadaf Zahra, Moiz Arshad Chohan

**Affiliations:** 1 M. Arshad Chohan, Department of Obstetrics & Gynaecology, Lady Willingdon Hospital / King Edward Medical University, Lahore, Pakistan; 2 Fauzia Butt, Department of Obstetrics & Gynaecology Sharif Medical & Dental College, Sharif Medical City Hospital, Lahore, Pakistan; 3 Maria Imran, Department of Obstetrics & Gynaecology, Lady Willingdon Hospital / King Edward Medical University, Lahore, Pakistan; 4 Sadaf Zahra, Department of Obstetrics & Gynaecology, Lady Willingdon Hospital / King Edward Medical University, Lahore, Pakistan; 5 Moiz Arshad Chohan Ghurki Trust Teaching Hospital, Lahore, Pakistan

**Keywords:** Placenta praevia, Accreta spectrum disorders, Postpartum haemorrhage, Peripartum hysterectomy

## Abstract

**Objectives:**

To describe the simplicity, efficacy and safety of A. Chohan Continuous Squeezing Suture (ACCSS) for controlling haemorrhage from the lower uterine segment at caesarean section for placenta praevia and accreta spectrum disorders.

**Methods:**

This prospective study was conducted on 47 patients with placenta praevia and accreta spectrum disorders from February 2019 to May 2022 in two teaching hospitals of Lahore and ACCSS was applied. The outcome measures were peripartum hysterectomy procedure time, estimated blood loss, number of blood transfusions, duration of stay in the hospital, bladder trauma, uterine necrosis, pelvic abscess formation, secondary postpartum haemorrhage and maternal mortality. Descriptive statistics were calculated by using SPSS version 21.

**Results:**

Out of 47 patients, 7 (15%) had placenta creta, 29 (61.7%) increta, 11 (23.3%) percreta (grade 3a), and 36 (76.6%) central anterior dominant placenta. Peripartum hysterectomy was prevented in 97.8% of patients. ACCSS procedure time was 5-10 minutes (87.2%), with mean blood loss 2500±485 ml, mean blood transfusion 1.85±1.02 units and mean hospital stay of 3.3±0.84 days. One patient had bladder trauma. There was no case of uterine necrosis, pelvic abscess formation, secondary postpartum haemorrhage or maternal mortality.

**Conclusion:**

ACCSS appears to be a simple, effective and safe treatment option for placenta praevia and accreta spectrum disorders, as an alternative to hysterectomy.

**Registration:** ClinicalTrial.gov NCT04660578, NCT05070689

## INTRODUCTION

Postpartum haemorrhage (PPH) has always been a prominent cause of maternal morbidity and mortality worldwide, with uterine atony as the leading cause and peripartum hysterectomy as the only definitive treatment option.^1^ Researchers have been struggling throughout the years to find a treatment modality other than hysterectomy, which has least morbidity and potential to preserve fertility. In an attempt to find a non-radical treatment for PPH due to uterine atony, B-Lynch introduced a novel compression suture. The time then witnessed emergence of a series of its variants from all over the world which effectively lowered the incidence of peripartum hysterectomy from uterine atony.^2^,^3^

Meanwhile the incidence of placenta praevia rose dramatically to 5.6 per thousand pregnancies and of abnormally adherent placenta to 24% after first and 67% after four or more caesarean sections.^3^ Both of these are attributable mainly to rising incidence of repeat caesarean sections.^4^ Placenta accreta spectrum disorders have opened up a new era in the history of PPH, as forcible separation of adherent placenta leads to massive bleeding from placental bed. Peripartum hysterectomy has once again emerged as gold standard treatment for this variety of PPH (three folds rise over uterine atony) with its overall morbidity of 40-50%, and mortality of 7-10% in case of placenta percreta.^5^ This high risk related to hysterectomy has led to the development of multiple conservative suturing techniques. Guang-Tai Li described three corner stones of uterine compression sutures i.e., simplicity, safety and efficacy and no such suturing technique so far is described in literature.^6^

Hence, it is the time for innovation in the treatment of placenta praevia and PAS. In this study, we report our own experience of a conservative surgical technique i.e. A. Chohan Continuous Squeezing Suture (ACCSS) application in the lower uterine segment during caesarean section in patients with placenta praevia and placenta accreta spectrum without involvement of urinary bladder and other pelvic organs. The objective of this study was to assess this surgical technique in the management of PAS, with the intention to develop a simple, safe and effective alternative to hysterectomy.

## METHODS

This multicenter prospective study was conducted in the departments of Obstetrics & Gynaecology of two tertiary care teaching hospitals in Lahore, Pakistan. Lady Willingdon Hospital, and Sharif Medical City Hospital Lahore, from February 2019 to May 2022 after having ethical approval from these institutes (497/RC/KEMU dated 12/02/2019, SMDC/SMRC/97-19 dated 27/06/2019, SMDC/SMRC/209-21 dated 11/8/2021) with registration from ClinicalTrials.gov NCT04660578, NCT05070689 (Annex-1). Fifty consecutively consenting patients, wishing to conserve the uterus, with ultrasound diagnosis of asymptomatic low-lying placenta and placenta praevia with placenta accreta spectrum disorder without bladder and other pelvic organs involvement (Grade-3a), were enrolled at 32 weeks of gestation by a purposive sampling technique.^7^ The patients with placenta accreta spectrum disorder with bladder and other pelvic organs involvement (Grade-3b & 3c), laterally situated placentae and those who required emergency caesarean section during study period were excluded.

At recruitment, the placental grading, site, and depth of placental invasion were determined at transvaginal sonography (TVS) by using 2D greyscale and 2D, 3D Doppler ultrasound. All ultrasounds were performed by two operators to reduce bias. The placenta lying directly over the internal os was labelled as praevia, and the one with leading edge within 20mm of internal os as low-lying placenta. The placental site was classified on the basis of its location into central (anterior dominant and posterior), and lateral (right and left).^8^ For placental invasion Unified ultrasound descriptors given in FIGO consensus guidelines were used.^9^

The recruited patients signed the informed consent form and were evaluated by detailed history and general and obstetrical examination. Demographic details and risk factors including maternal age, parity, duration of gestation, spontaneous or assisted conception and previous history of placenta praevia, myomectomy, caesarean sections, endometrial curettage, and resection of uterine septum or intrauterine adhesions were recorded. The patients were given corticosteroids at 34 weeks. Haemoglobin was assessed and corrected with parenteral iron/blood transfusion. The patients were admitted at 36 weeks and counselled about the details of surgical procedure, choice of anaesthesia, risks of massive obstetric haemorrhage, lower urinary tract damage, peripartum hysterectomy and need for blood transfusions.

The caesarean sections were performed between 37 and 38 weeks of gestation on all patients. Haematology department was placed on alert with availability of four units of cross matched fresh blood. All surgeries were performed by principal investigators (Prof. M. Arshad Chohan & Prof. Fauzia Butt). A multidisciplinary team comprising of haematologist, anaesthetist and paediatrician was involved during the procedure. Prophylactic antibiotic (ceftriaxone 1gm intravenously) was administered to all patients before surgery.

At caesarean section Pfannenstiel incision was used. A transverse incision was given in the lower uterine segment (LUS) above the insertion of placenta and the baby was delivered. At delivery of baby ten IU of oxytocin were given intravenously, followed by 40 IU in 500 ml of normal saline at the rate of 125 mililiter/hour for first 24 hours. The uterus was exteriorized without making any efforts to remove the placenta. Bladder was dissected away only where it was adherent with the lower uterine segment from previous caesarean sections. Uterine arteries were ligated on both sides and any blood vessels on the way were secured immediately. The placenta was then forcibly removed to as close to complete as possible. The lower uterine segment was packed with sponge to arrest haemorrhage temporarily while preparing for the suture.

**Fig.1:**
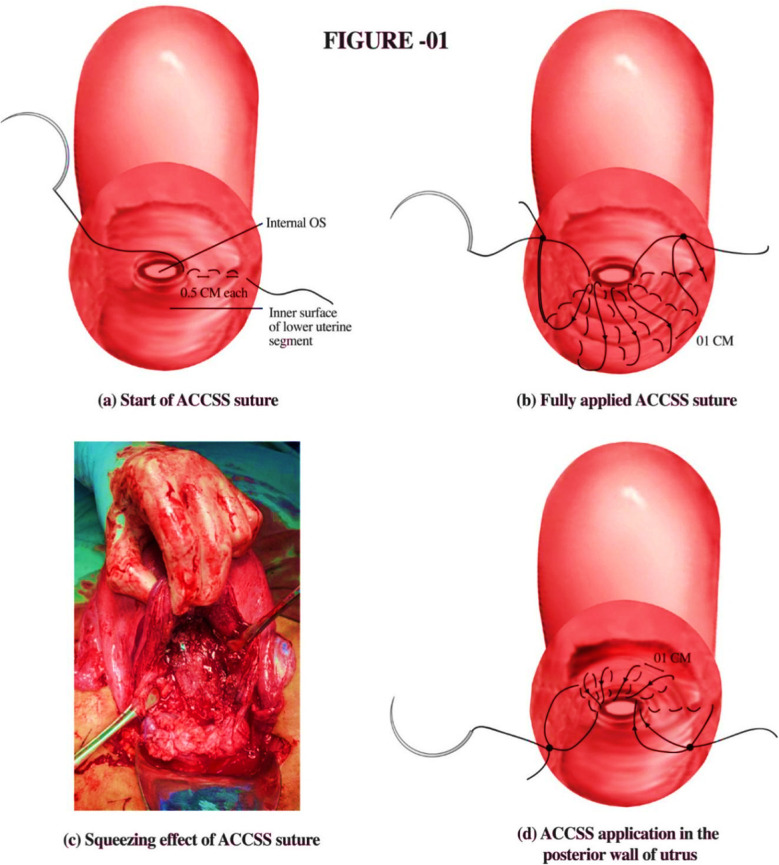
A. Chohan Continuous Squeezing Suture (ACCSS) application at caesarean section for placenta praevia/accreta ***Suture material:*** Half circle 40 mm round body polyglactin 910 suture # 1 (vicryl plus by Ethicon ®) ***Suture application:*** (i). Multiple half cm bites are taken at half cm interval through half-thickness of the tissue starting from the outer edge of lower uterine segment incision and running through its inner surface to reach the outer half of ring of internal os. (ii). The suture is then tied and first knot secured causing squeezing of uterine tissue. (iii). Similar sutures are placed continuously at 1-cm distance till the right corner is reached, where the second knot is secured. (iv). Suture on the posterior uterine wall is started from the outer half of the posterior lip of the ring of internal os and going up to the highest bleeding point on the posterior uterine wall, continuing from the left to right end of the uterine incision

The packing was removed and within the puddle of blood, the ring of internal os was identified with the index and middle finger of one hand and held with Babcock forceps with the other hand. On the exposed inner surface of the LUS, suturing was started from the left corner of uterine incision, using half circle 40 millimeter round body polyglactin 910 suture#1 (Vicryl plus by Ethicon ®) taking multiple half centimeter bites through half-thickness of the tissue at half centimeter intervals to reach the outer half of ring of internal os. The suture was then tied and first knot secured causing squeezing of uterine tissue. From here onwards similar sutures were placed continuously at one cm distance till the right corner was reached, where the second knot was secured. During suturing it was ensured that internal os remains patent. The continued pull on the suture caused the squeezing of the LUS and arrested bleeding from all sinuses present at the placental site. If the bleeding was seen on the posterior uterine wall, a similar suture was applied. It was started from the outer half of the posterior lip of the ring of the internal os and going up to the highest bleeding point on the posterior wall of the uterus, continuing from the left to right end of the uterine incision (Fig.1). Any leftover bleeding points were secured with separate sutures to ensure complete haemostasis. The uterine incision was closed in two layers as done in a routine lower segment caesarean section. Any additional medical or surgical therapy instituted was recorded.

Gravimetric method was used for calculation of blood loss during surgery.^10^ The pre and postoperative difference of weight of swabs and sponges was taken as one ml=1g. Blood collected in the suction bottles was recorded. Pooled blood on surfaces was estimated as 50cm=500 ml, 75 cm=1000 ml, and 100 cm=1500 ml. Fist size blood clot=50g. Blood transfusion necessity was determined by assessment of blood loss and mean drop in haemoglobin concentration.

Most of the patients were discharged from hospital on day three with some needing extra stay up till 7^th^ day. During hospital stay vital signs, vaginal bleeding, lochia and wound status were checked on regular basis. All patients were reviewed on 7^th^ postoperative day and at six weeks postpartum. History of fever, abdominal pain, and excessive vaginal bleeding, and lactation / menstruation status were recorded. Pelvic ultrasound was done on both visits to detect any pelvic organ abnormality and collection of blood or pus.

The efficacy of ACCSS was measured as the number of peripartum hysterectomies, simplicity as procedure time (application time of suture plus time to complete haemostasis), and safety was measured as estimated blood loss, number of blood transfusions, duration of stay in the hospital, bladder trauma, uterine necrosis, pelvic abscess formation, secondary postpartum haemorrhage and maternal mortality.

Study data was analysed using Statistical Package Software for Social Sciences, IBM, USA (SPSS version 21). Descriptive statistics were calculated by using mean and standard deviation for continuous variables, and frequency and percentages for categorical variables.

## RESULTS

A total of 50 patients were recruited. Three patients required emergency caesarean section for preterm labor with heavy vaginal bleeding and were excluded from study, hence the results of 47 patients are being reported here. Six (12.8%) patients had low lying placenta, while 41 (87.2%) had placenta praevia. Among 41 patients with placenta praevia 36 (87.8%) had anterior dominant, and five (12.2%) had posterior placenta. Out of 47 patients seven (15%) were with placenta creta, twenty nine (61.7%) with placenta increta and eleven (23.3%) had placenta percreta (grade 3a). The mean age of patients was 31.78±4.1 years, mean duration of gestation was 37±0.46 weeks and the mean parity was 3.1±1.20. Twenty-three (48.9%) patients had previous three caesarean sections (Table-I). Fourteen patients (29.8%) had previous history of endometrial curettage. All patients had spontaneous conception with no history of myomectomy, resection of uterine septum or intrauterine adhesions.

**Table-I:** Demographic details of ACCSS.

Variables	Frequency N=47	Percentage %
***Age (Years)***		
18-25	3	6.4
26-30	17	36.2
31-35	19	40.4
>35	8	17.0
***Previous caesarean section***		
None	1	2.1
One	5	10.6
Two	9	19.1
Three	23	48.9
Four and more	9	19.1
***Placental Grade***		
Low lying	6	12.8
Placenta praevia	41	87.2
***Placental Location***		
Placenta praevia (central)	36	76.6
Anterior dominant	5	10.6
Posterior	6	12.8
Low lying (anterior)		
***Placenta Accreta Spectrum (PAS)***		
Creta	7	15
Increta	29	61.7
Percreta (Grade 3 a)	11	23.3

Out of 47 patients receiving ACCSS one patient (2.1%) needed peripartum hysterectomy for concomitant uterine atony. Complete haemostasis at ACCSS procedure was achieved in 5-10 minutes in 41 (87.2%) patients and 11-15 minutes in six (12.8%) patients. The estimated blood loss ranged from 1500 ml to 4000 ml with a mean loss of 2500±485 ml. The mean blood transfusion was 1.85±1.02 units. The mean drop in haemoglobin concentration was 1.3 g/dl (10.6±0.52 g/dl preoperatively versus 9.28±1.48 g/dl postoperatively). One patient (2.1%) had bladder injury needing repair (Table-II).

**Table-II:** Outcome Measures of ACCSS.

Outcomes	Frequency N=47	Percentage%
***Time Duration of ACCSS Procedure (min)***		
5-10 min	41	87.2
11-15min	6	12.8
***Estimated blood loss at surgery (ml)***		
<2000	10	21.3
2000-3000	33	70.2
>3000	4	8.5
***Isolated Hematoma formation***		
Yes	12	2 5.5
No	35	74.5
***Injury to bladder***		
Yes	1	2.1
No	46	97.9
***No. of Blood Transfusions (Units)***		
One	18	38.3
Two	20	42.5
Three	7	14.9
Four and more	2	4.3
***Duration of Stay in Hospital (days)***		
1-3	39	82.9
4-6	7	15.0
>7	1	2.1
***Number of hysterectomies***		
Yes	1	2.1
No	46	97.8

Twelve (25.5%) patients developed a small intraoperative haematoma around the suturing site in the LUS that was managed by separate haemostatic sutures. No patient required additional conservative surgical treatment. Ten patients were given an additional dose of five i.u. of oxytocin plus 500 mg tranexamic acid intravenously. The mean duration of stay in the hospital was 3.3±0.84 days. No patient had any evidence of ischaemia, abscess or haematoma formation, or secondary PPH. There was no maternal mortality.

## DISCUSSION

Lower uterine segment has a complex blood supply which is further complicated by the neovasculature of placenta accreta. Additionally, LUS has less smooth muscle component, which compromises its retraction ability and squeezing power to control blood loss. Therefore, the placental site in the lower uterine segment bleeds profusely on separation of placenta especially when placenta praevia is also accreta. To avoid such catastrophe Sentilhes introduced the term conservative treatment of placenta accreta which was then called expectant management or leaving placenta in situ approach. This approach avoids such bleeding and has shown to avoid hysterectomy in 78% of cases but is associated with an extensive range of morbidities.^11^ Triple-P procedure and one step conservative surgery also advocates non-separation of placenta and removal of myometrium at placental site with intact placenta. These procedures involve extensive pelvic devascularization and expensive radiological setups. The former includes tubal ligation as well hence affecting the future fertility.^12^,^13^

The studies describing the separation of placenta have relied upon pelvic devascularization or using an inverted cervix as a tamponade but none of them has described the actual suturing technique of the bleeding site. The cervical tamponade procedures also cause anatomical distortion, though described as temporary.^7^,^14^

ACCSS is a novel suturing technique which addresses the management of placenta praevia and PAS following the orthodox approach of separation of placenta, taking the challenge of controlling massive haemorrhage. The rationale of ACCSS is based upon the following: The lower uterine segment is thin, flexible, squeezable and holds the suture well. Internal cervical os is a fixed structure, and has a ring with sufficient strength to function as anchor to the suture. The bleeding area on inner surface of LUS upon separation of placenta does not extend onto the internal os and into the vagina. Taking half thickness of internal cervical os into suture does not alter the anatomy and uterine drainage remains unaffected. Bilateral uterine artery ligation is added to it because occlusion of the uterine artery or its branches is useful procedure to stop upper uterine bleeding, it does not appear to affect fertility or obstetric outcome and vascular occlusion is only temporary, as recanalization soon ensures normal uterine circulation.^15^ The haemostatic effect of ACCSS is therefore independent and does not rely upon supportive measures.

In this study ACCSS was successful in preventing hysterectomy in 97.8% of patients. It is quicker than the procedures described by El Gelany, Meng, JC Shih.^16^-^18^ The estimated procedure related blood loss and blood transfusion is well within the reported range.^7^,^19^,^20^ The woman needing hysterectomy was multiparous with previous four caesarean sections, central anterior dominant placenta praevia increta and concomitant uterine atony. She was the patient who had bladder injury, blood loss 4000 ml and was transfused six units of blood.

Cho compression suture brings haemostasis by compression of anterior and posterior uterine walls together and causes closure of the uterine cavity but is associated with hematoma, abscess and intra uterine adhesion formation, while Nausicaa suture is reported to cause ischemia of the uterine wall.^18^ No such complications were recorded after ACCSS. In our study, no additional conservative surgical treatment was instituted to any patient. There was no case of secondary postpartum haemorrhage or maternal mortality.

### Strengths & Limitations:

The hallmark of the suture is its simplicity and that too without the use of extensive interventions or expensive equipment, as well as expanded multidisciplinary involvement. ACCSS involves suturing in a field full of blood which demands operator to be swift and in self-control. Generally, first 3-4 serial sutures bring the severity of haemorrhage under control and rest of the procedure can be carried out with relative ease. In patients with haematoma formation, the complication can be prevented by reducing the inter suture distance. Isolated hematomas are usually small and can be dealt with easily by additional haemostatic sutures.

Limited sample size and an adequately high-powered randomized control trial would be required to enhance the validity of this surgical technique, and to assess its suitability for placenta percreta beyond Grade-3a.

## CONCLUSION

ACCSS is a simple, safe and effective suture, has a short learning curve, is easily reproducible and provides a practicable alternative to hysterectomy without the need for extensive and expensive interventional radiological setups.

### Authors’ Contributions:

**AC**: Conceived the idea, project, design and development, manuscript writing, editing, revision of final manuscript and is responsible for integrity of research.

**FB**: Project design and development, manuscript writing and editing, data development and analysis.

**MI**: Data development, manuscript writing.

**SZ**: Data collection and data development.

**MC**: Data collection, recruitment and follow up of patients.

All authors read and approved the final manuscript.

## References

[1] Dohbit JS, Foumane P, Nkwabong E, Kamouko CO, Tochie JN, Otabela B (2017). Uterus preserving surgery versus hysterectomy in the treatment of refractory postpartum haemorrhage in two tertiary maternity units in Cameroon:A cohort analysis of perioperative outcomes. BMC Pregnancy Childbirth.

[2] Jiang H, Wang L, Liang J (2020). Uterine comression suture is an effective mode of treatment of postpartum haemorrhage. Pak J Med Sci.

[3] Jauniaux E, Grønbeck L, Bunce C, Langhoff-Roos J, Collins SL (2019). Epidemiology of placenta previa accreta:A systematic review and meta-analysis. BMJ Open.

[4] Wasim T, Bushra N, riaz S, Iqbal HI (2020). fetomaternal outcome in patients with placenta previa. Pak J Med Sci.

[5] Huque S, Roberts I, Fawole B, Chaudhri R, Arulkumaran S, Shakur-Still H (2018). Risk factors for peripartum hysterectomy among women with postpartum haemorrhage:Analysis of data from the WOMAN trial. BMC Pregnancy Childbirth.

[6] GLXLB Wu, Xu GLH (2015). Three cornerstones of uterine compression sutures :simplicity, safety and efficacy. Arch Gynecol Obstet.

[7] Shabana A, Fawzy M, Refaie W (2015). Conservative management of placenta percreta:a stepwise approach. Arch Gynecol Obstet.

[8] Jauniaux ERM, Alfirevic Z, Bhide AG, Belfort MA, Burton GJ, Collins SL (2019). Placenta praevia and Placenta Accreta:Diagnosis and Management:Green-top Guideline No. 27a. BJOG An Int J Obstet Gynaecol.

[9] Sentilhes L, Kayem G, Chandraharan E, Palacios-Jaraquemada J, Jauniaux E, Duncombe G (2018). FIGO consensus guidelines on placenta accreta spectrum disorders:Conservative management. Int J Gynecol Obstet.

[10] Vitello DJ, Ripper RM, Fettiplace MR, Weinberg GL, Vitello JM (2015). Blood Density Is Nearly Equal to Water Density:A Validation Study of the Gravimetric Method of Measuring Intraoperative Blood Loss. J Vet Med 2015.

[11] Sentilhes L (2018). Conservative Management of placenta Accreta Spectrum. Clin Obstet Gynaecol.

[12] Abbas Riwa A, Nassar,Anwar H (2021). Placenta accreta spectrum:consrvative management and Its Impact on Future Fertility. Maternal-Fetal Medicine.

[13] Palacios-Jaraquemada JM, Fiorillo A, Hamer J, Martinez M, Bruno C (2022). Placenta accreta spectrum:a hysterectomy can be prevented in almost 80% of cases using a resective-reconstructive technique. J Matern Neonatal Med.

[14] Sakhavar N, Heidari Z, Mahmoudzadeh-sagheb H (2018). International Journal of Gynecology and Obstetrics Cervical inversion as a novel technique for postpartum hemorrhage management during cesarean delivery for placenta previa accreta / increta. Int J Gynecol Obstet.

[15] AbdRabbo SA (1994). Stepwise uterine devascularization:A novel technique for management of uncontrollable postpartum hemorrhage with preservation of the uterus. Am J Obstet Gynecol.

[16] El Gelany SAA, Abdelraheim AR, Mohammed MM, Gad El-Rab MT, Yousef AM, Ibrahim EM (2015). The cervix as a natural tamponade in postpartum hemorrhage caused by placenta previa and placenta previa accreta:A prospective study. BMC Pregnancy Childbirth.

[17] Meng Y, Wu P, Deng D, Wu J, Lin X, Beejadhursing R (2017). Multifaceted spiral suture:A hemostatic technique in managing placenta praevia or accrete. Med (United States).

[18] Shih JC, Liu KL, Kang J, Yang JH, Lin MW, Yu CU (2019). 'Nausicaa'compression suture:a simple and effective alternative to hysterectomy in placenta accreta spectrum and other causes of severe postpartum haemorrhage. BJOG.

[19] Majeed T, Waheed F, Mahmood Z, Saba K, Mahmood H, Bukhari MH (2015). Frequency of placenta previa in previously scarred and non scarred uterus. Pak J Med Sci.

[20] Khaliq Showman HA, Alizzi FJ, Helmi ZR, Ismael VA, Fawzi HA (2019). Placenta accrete spectrum disorders:A single centre experience over four years in the view of international guidelines. J Pak Med Assoc.

[21] Makwana Shailesh K, Sonal C, Chirag P (2020). Uterine preservation with Cho suture in localized multifocal atonicity of uterus with failed medical management and uterine tamponade. Int J Reprod Contracept Obstet Gynecol.

